# Maximum likelihood estimation of difference scaling functions for suprathreshold judgments

**DOI:** 10.1167/jov.22.10.9

**Published:** 2022-09-09

**Authors:** Emily S. Teti, Terece L. Turton, Jonah M. Miller, Roxana Bujack

**Affiliations:** 1 Computer, Computational, and Statistical Sciences Division, Los Alamos National Laboratory, Los Alamos, NM, USA; 2 Department of Psychology, Florida Atlantic University, Boca Raton, FL, USA

**Keywords:** maximum likelihood estimation, difference scaling, suprathreshold difference judgments

## Abstract

Maximum likelihood estimation (MLE) has been used to produce perceptual scales from binary judgments of triads and quadruples. This method relies on Thurstone's theory of a stochastic perceptual process where the perceived difference of two stimuli is the difference in their perceived strengths. It is possible that the perception of a suprathreshold difference is overestimated when adding smaller differences, a phenomenon referred to as diminishing returns. The current approach to construct a perceptual scale using MLE does not account for this phenomenon. We present a way to model the perception of differences using MLE and Thurstone's theory, adapted to allow the possibility of diminishing returns. This method is validated using Monte Carlo simulated responses to experimental triads and can correctly model diminishing returns, the absence of diminishing returns, and the opposite of diminishing returns both in the cases when a perceptual scale is known and when the true perceived strengths of the stimuli are unknown. Additionally, this method was applied to empirical data sets to determine its feasibility in investigations of perception. Ultimately, it was found that this analysis allows for more accurate modeling of suprathreshold difference judgments, a more complete understanding of the perceptual processes underlying comparisons, and the evaluation of Thurstone's theory of difference judgments.

## Introduction

For close to a century, the ability to construct perceptual scales of stimulus strengths from two-alternative forced-choice (2AFC) experimental tasks has relied on Thurstone's law of comparative judgment (Thurstone, 1927). This theory, referred to as *Thurstonian scaling*, states that the perception of a stimulus is normally distributed about its true perceived strength. This extends to the perceived differences of stimuli as the difference of normally distributed variables is also normally distributed. However, the universality of this assumption across different perceptual variables has not been sufficiently validated and cannot be currently verified using tools that assume Thurstonian scaling.

The assumption of Thurstonian scaling allowed for the development of powerful tools for multidimensional scaling (MDS), such as Torgerson's method of MDS (Torgerson, 1952). These tools have been implemented as software packages that are easily accessible for behavioral scientists (Kruskal, 1964; Young & Torgerson, 1967; Carroll & Chang, 1970; Busing et al., 1997). Later work used maximum likelihood estimation (MLE) to estimate perceptual scales from experimental results based on Thurstone's Case V (Knoblauch et al., 2008; Maloney & Yang, 2003). Thurstonian scaling has been used for decades to construct perceptual scales of social attitudes (Messick, 1956), personality (Jackson et al., 1957), and, more recently, image compression (Charrier et al., 2007), organization of scattered points (Protonotarios et al., 2016), and color (Bonnardel et al., 2016).

Thurstone's theory allows for the construction of a scale of stimulus strengths from 2AFC tasks by using the *z*-score of the proportion of responses choosing one test over the other. Consider a triad arrangement of three stimuli, a standard and two tests referred to as the method of triads (MOT). These will be referred to as $S_{stand},S_{test1},S_{test2}$. The participant is asked to determine which test is *more different* from the standard. Under Thurstonian scaling assuming Thurstone Case V, the perception of these stimuli, S, are normally distributed variables centered on their true perceived strength, ψ, with a variance, $\sigma^{2}$, which Thurstone names *discriminal dispersion*,

(1) $$\begin{array}{c} \begin{array}{cc} \mathrm{Percept}(S_{stand}) & ∼N(\psi_{stand},\sigma^{2})\\ \mathrm{Percept}(S_{test1}) & ∼N(\psi_{test1},\sigma^{2})\\ \mathrm{Percept}(S_{test2}) & ∼N(\psi_{test2},\sigma^{2}). \end{array}\; \end{array}$$

If triads are constrained such that the standard is always stronger than test1 and weaker than test2, the judgment, “Identify which difference is larger,” can be written as

(2) $$\begin{array}{ccc} \;\;\;\;\;\;\;\;\; & & \;\;\;\;\;\;\;\;\;\mathrm{Percept}(\left(S_{stand}-S_{test1}\right)-\left(S_{test2}-S_{stand}\right))\\ & & \;\;\;\;\;\;\;\;\;\;∼N(\left(\psi_{stand}-\psi_{test1}\right)-\left(\psi_{test2}-\psi_{stand}\right),4\sigma^{2}).\; \end{array}$$

The comparison of this variable to 0 gives the probability that a participant would select test1 over test2,

(3) P(SelectingTest1)=P(Percept((Sstand-Stest1)P(SelectingTest1)=-(Stest2-Sstand))>0)=12σ2π∫-∞(ψstand-ψtest1)-(ψtest2-ψstand)e-x24σ2dx.

The final line from Equation (3) is the cumulative normal distribution. For the remainder of this article, the cumulative normal distribution will be referred to as

(4) $$\Phi_{\sigma }\left(t\right)=\frac{1}{2\sigma \sqrt{2\pi }}\int_{-\infty }^{t}e^{-\frac{x^{2}}{4\sigma^{2}}}dx.$$

This method is straightforward and easy to implement. Knoblauch et al. (2008) created an open-source package to carry out this analysis for MOT and method of quadruples (MOQ). This analysis has been used on both MOT and MOQ studies to investigate many quantifiable characteristics of visual stimuli (e.g. Devinck & Knoblauch, 2012; Aguilar et al., 2017). However, it does assume that the perception of the differences can be added and subtracted as normally distributed variables. We refer to this as the *additivity of differences*. This additivity is inherent to the Thurstonian model even though it may or may not exist in reality. More important, relying on an analysis methodology that assumes additivity precludes evaluation of its existence.

**Figure 1.**
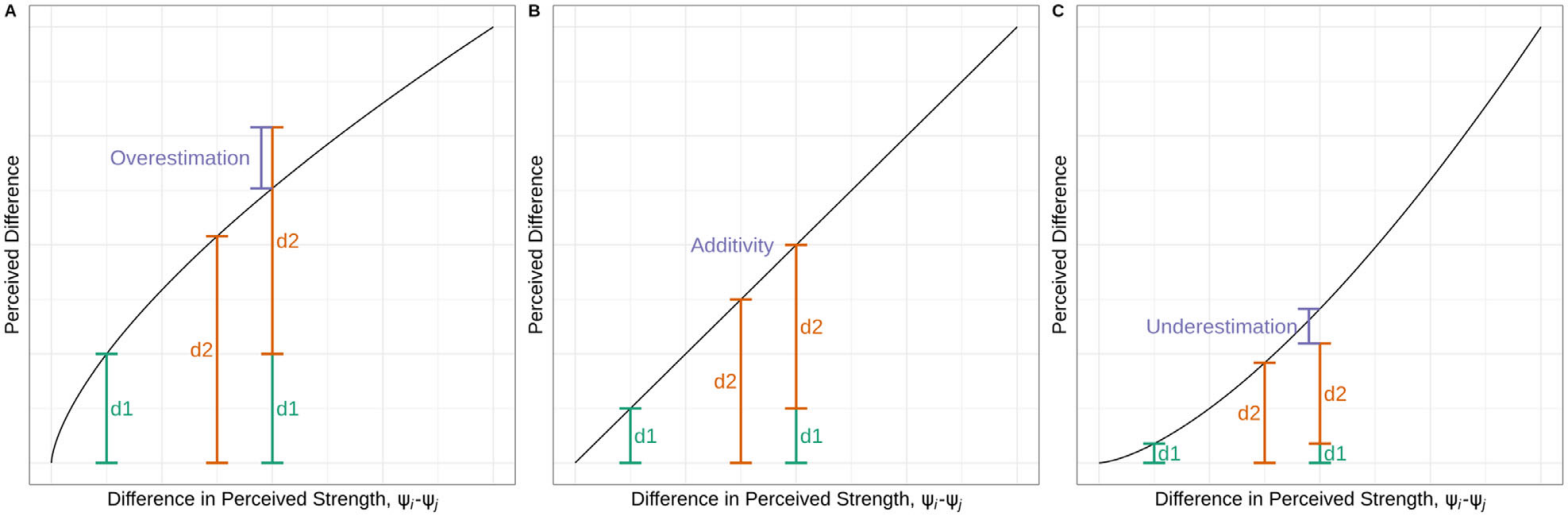
The relationship between concavity and diminishing returns. Diminishing returns can be described as a concave scaling function that scales $\psi_{i}-\psi_{j}$ to give the perceived difference, as seen in (A). If small differences are additive to equal large differences, the scaling function would be linear, as in (B). If increasing returns exist, the perceived difference would have a convex relationship with $\psi_{i}-\psi_{j}$, seen in (C).

If the additivity of differences does not exist, that would imply that larger differences are not the sum of smaller differences. Should the addition of small differences overestimate the perception of a large difference, this would be a case of *diminishing returns*, similar to a second-order Weber–Fechner law. This behavior can be modeled as a function that takes in a difference in ψ and returns the perceived difference using a concave function. If the addition of small differences underestimates the perception of a large difference, this would be a case of *increasing returns*. This would indicate that a scaling function would be convex. Illustrative scaling functions for the cases of diminishing returns, additivity of differences, and increasing returns are shown in Figure 1.

In the cases of diminishing and increasing returns, analysis methods that rely on Thurstonian scaling will result in a perceptual scale that does not take this behavior into account. As such, they cannot be used to evaluate the existence of diminishing or increasing returns. The perception of varying sizes of differences has been studied previously in both color perception (Bonnardel et al., 2016) and organization of points in a field (Protonotarios et al., 2016), but results were inconclusive, likely due to the reliance on analysis methods that assume additivity. These results are unsurprising as it has been explicitly stated that “the only controversy that might be relevant [to the use of MLE for difference scaling] is whether the difference measurement model is an adequate model of human judgement of suprathreshold perceptual differences” (Maloney & Yang, 2003).

The present work offers a solution to the additivity assumption by directly modeling the underlying scaling function using MLE without making assumptions about its shape. This work builds on previous analysis that uses MLE to construct a perceptual scale (Maloney & Yang, 2003; Knoblauch et al., 2008), but it is a step away from strict Thurstonian scaling. This approximation tool would offer a solution to those investigating how small and large differences are perceived in any characteristic that is easily quantified.

## Maximum likelihood estimation for difference scaling

MLE is used to estimate parameters of a stochastic process using a sample of observations. MLE optimizes the likelihood of the estimated parameters given the data,

(5) $$\begin{array}{c} L(\theta |y)=P(y|\theta )\; \end{array}$$

where $y=y_{1},y_{2},...,y_{n}$ is an independent, identically distributed sample of size n; $\theta =\theta_{1},\theta_{2},...,\theta_{m}$ is the set of parameters to estimate that describes the distribution of $y_{i}$; and P(y|θ) is the joint probability of seeing the sample given the set of parameters (Myung 2003). This function can be optimized analytically if L(θ|y) can be differentiated with respect to θ. Otherwise, the optimal values can be approximated numerically. The latter is the case for the MLE approach used in this study. Since optimal values are found numerically, which can result in local optima, 10 iterations of the optimization are performed with different, random initial parameters.

In practice, the logarithm of L(θ|y), or the log likelihood, is optimized so probabilities are summed rather than multiplied to calculate the joint probability. Here, we use an optimizer that minimizes, so we negate the log-likelihood to define the negative log-likelihood as our objective function.

### Maximum likelihood for difference modeling

It has been established that participant responses can be modeled using a cumulative Gaussian, $\Phi_{\sigma }\left(x\right)$, centered at 0 with a variance, $\sigma^{2}$ (Maloney & Yang, 2003). Using the perceived strengths of stimuli $S_{stand}$, $S_{test1}$, $S_{test2}$, the probability of a given response is defined as

(6) $$\begin{array}{ccc} & & \;\;\;\;\;\;\;\;\;\;\;\;P\left(R|S_{stand},S_{test1},S_{test2}\right)=\Phi_{\sigma }(\left(\psi_{stand}-\psi_{test1}\right)\\ & & \;\;\;\;\;\;\;\;\;\;\;\;\;-\;\left(\psi_{test2}-\psi_{stand}\right){)}^{R}*(1-\Phi_{\sigma }(\left(\psi_{stand}-\psi_{test1}\right)\\ & & \;\;\;\;\;\;\;\;\;\;\;\;\;-\;\left(\psi_{test2}-\psi_{stand}\right){))}^{1-R},\; \end{array}$$

where R=1 indicates that participants selected the first test, test1, as the most different test in the triad and R=0 otherwise. This equation relies on σ, the standard deviation of the Gaussian, which can be estimated along with the $\psi_{i}$ using MLE (Knoblauch et al., 2008; Maloney & Yang, 2003). The standard deviation is constant for every $\psi_{i}$, consistent with Thurstone's Case V (Thurstone, 1927). This analysis has been written into an open-source package, MLDS, for use in the statistical programming language, R (Knoblauch et al., 2008), as well as MATLAB and Python (Maloney & Knoblauch, 2020).

In order to capture any effect of diminishing or increasing returns, the sizes of the differences need to be scaled in a possibly nonlinear way. In the following, we represent the perceived difference between stimuli $S_{i}$ and $S_{j}$ as

(7) $$\begin{array}{c} Percept\left(S_{i}-S_{j}\right)=f(|\psi_{i}-\psi_{j}\left|\right).\; \end{array}$$

Here, f is the scaling function responsible for diminishing or increasing returns. This function can be approximated using MLE. The approximation, $\hat{f}$, can be incorporated into Equation (6). The resulting stochastic model takes the form

(8) $$\begin{array}{ccc} & & \;\;\;\;\;\;\;\;\;\;\;\;\;\;\;P\left(R|S_{stand},S_{test1},S_{test2},\hat{f}\left(x\right)\right)=\Phi_{\sigma }(\hat{f}\left(\right|\psi_{stand}-\psi_{test1}\left|\right)\;\;\;\;\\ & & \;\;\;\;\;\;\;\;\;\;\;\;\;\;\;\;-\hat{f}\left(\right|\psi_{stand}-\psi_{test2}{\left|)\right)}^{R}*(1-\Phi_{\sigma }(\hat{f}\left(\right|\psi_{stand}-\psi_{test1}\left|\right)\;\;\;\;\\ & & \;\;\;\;\;\;\;\;\;\;\;\;\;\;\;\;-\hat{f}\left(\right|\psi_{stand}-\psi_{test2}{\left|))\right)}^{1-R}. \end{array}$$

This represents the likelihood of a single response, R, in a given triad. To perform the MLE, the sum of the log of the value of Equation (8) for each response will be maximized with respect to parameters that determine $\hat{f}$. This MLE was written using the optimization function, *optim*, in R (R Core Team, 2021). This function minimizes, so the final form of the objective function is the negative log-likelihood (NLL),

(9) $$\begin{array}{c} \;\;\;\;\;\;\;\;\;\;\;\;NLL=-\sum_{\forall t,n}log(P\left(R^{t,n}|S_{stand}^{t,n},S_{test1}^{t,n},S_{test2}^{t,n},\hat{f}\left(x\right)\right)\; \end{array}$$

where the superscript, t, indicates the specific triad and the superscript, n, indicates the participant. It should be noted that NLL is not normalized by the number of participants or trials. When comparing the NLL from models built with different amounts of one or both, the NLL should be divided to give the average NLL.

It may be possible to model a discrete mapping of the perceived differences, $d_{1}^{'},d_{2}^{'}$, from every value of $d_{1}=\psi_{stand}-\psi_{test1},d_{2}=\psi_{test2}-\psi_{stand}$ if we assume that the stimuli used are selected in equal step sizes, for instance, each stimulus is one just noticeable difference (JND) from the previous. If, however, there are many different values of $d_{1},d_{2}$, or the stimuli are not equally spaced, the underlying scaling function should be modeled using a continuous function, as there would be insufficient data to estimate a large number of parameters. In both cases, a monotonicity constraint will be placed on the model such that if $d_{1}<d_{2}$, then $d_{1}^{'}<d_{2}^{'}$. In the case of modeling a continuous function, it will also be necessitated that $\hat{f}$ will be continuous and differentiable everywhere, which can be achieved by a spline function.

A spline function is a piecewise polynomial that is differentiable everywhere (Epstein, 1976). Specifically, a cubic, monotonic Hermite spline using the Fritsch and Carlson method is used in the MLE approximation of f (Fritsch & Carlson, 1980). Each cubic segment can be represented as

(10) $$\;\;\;\;\;\;\;\;\;\;\;\;sp\left(x\right)=\left\{\begin{array}{c} \beta_{0}^{a}+\beta_{1}^{a}x+\beta_{2}^{a}x^{2}+\beta_{3}^{a}x^{3},0<x\leq a_{x}\\ \beta_{0}^{b}+\beta_{1}^{b}x+\beta_{2}^{b}x^{2}+\beta_{3}^{b}x^{3},a_{x}<x\leq b_{x}\\ \beta_{0}^{c}+\beta_{1}^{c}x+\beta_{2}^{c}x^{2}+\beta_{3}^{c}x^{3},b_{x}<x\leq c_{x} \end{array}\right..\;\;\;\;\;\;\;\;\;$$

### Hypothesis testing for nonadditivity

Traditional hypothesis testing can be applied to check if additivity can be assumed. If color differences are additive, the perception of the difference of differences, $\Delta d=d_{1}-d_{2}$, should not depend on the size of the individual differences. It follows that participant accuracy should not be affected by the average size of differences in a triad, $\bar{d}=0.5*\left(d_{1}+d_{2}\right)$. In this case, a linear regression,

(11) $$\hat{acc}=h\left(\Delta d,\bar{d}\right)=\beta_{0}+\beta_{1}\left|\Delta d\right|+\beta_{2}\bar{d},$$

built to include this term should return a $\beta_{2}$ coefficient close to zero. A coefficient for the average difference term significantly different from zero supports the claim that small differences are not additive for equivalent large differences. This can be formalized as a pair of null and alternative hypotheses,

(12) $$\begin{array}{c} \begin{array}{c} H_{0}:\beta_{2}=0,\\ H_{1}:\beta_{2}\neq 0, \end{array}\; \end{array}$$

where the alternative hypothesis is the general case of nonadditivity. In the case of diminishing returns, accuracy is expected to decrease with increasing average difference, $\bar{d}$, resulting in a significantly negative coefficient. In the case of increasing returns, the opposite is expected. For the purposes of this study, we determine significance using a *t*-test, which is conveniently built into the regression in R. The hypothesis testing is meant as a preliminary analysis as it makes the strong assumption that the perceptual scale is well known. This assumption is likely to fail, even if a perceptual scale has previously been estimated assuming additivity. However, in application, other statistical tests can be used to determine whether the regression suggests nonadditivity such as a likelihood ratio test.

## Validation study assuming a known perceptual scale

To validate the analysis methods detailed above, they were applied to simulated data where the true underlying function is known. These simulations were carried out under a number of conditions describing the underlying perceptual process and the choice of MLE model to use. In all cases, the hypothesis testing is applied for the purpose of investigating the significance of the coefficient for the average difference term. The MLE-approximated $\hat{f}$ is evaluated using both the negative log-likelihood used in optimization and the root mean square error (RMSE) of the approximated function and ground truth.

### Methods

A number of factors in the data generation and MLE fit were varied to investigate the strengths and limitations of the proposed analysis. A summary of these factors is included in Table 1.

**Table 1.** Description of the variables manipulated for the simulation study to validate the proposed analysis.

Variable	Description	Values
N	Number of responses per triad	25, 50, 100, 200, 300
$\sigma_{DD}$	True discriminal dispersion	3.63, 8.03, 12.41, 16.79
f	True underlying scaling function	Concave: circle, root, sine linear convex
$\hat{f}$ structure	Number of parameters describing approximated function	Spline with 4 parameters spline with 8 parameters discrete with 12 parameters
Scale max	Size of estimated perceptual scale	Maxima set to: 1.2, 12, 120; Standard deviation set to: 0.146, 1.46, 14.6

#### Experimental triads

**Figure 2.**
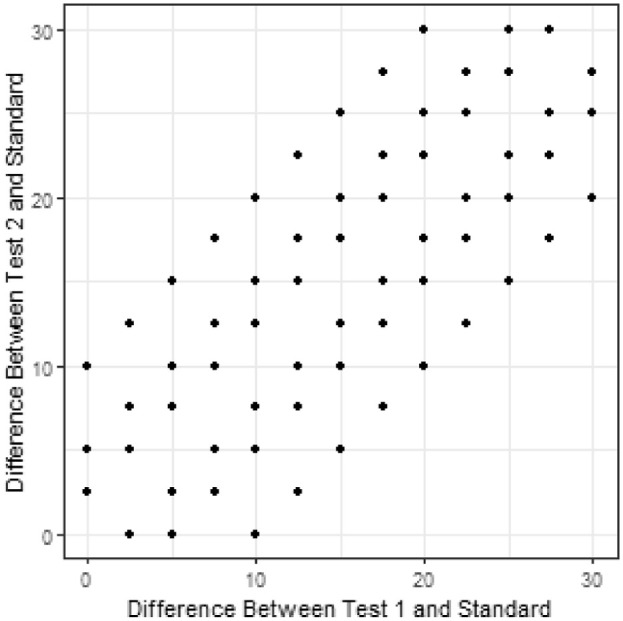
Representation of the triads used for simulation. Each point represents combinations of $d_{1}$ and $d_{2}$ tested.

The same experimental design was used in all simulated cases. This simulation assumes an experimental design used to investigate the perception of varying sizes of differences in a quantifiable characteristic, S. For the purpose of this simulation, S∈[0,100]. Differences in S included in the experimental design range from 0 to 30 in steo sizes of 2.5 to give 12 non zero differences investigated. Difference in differences, Δd, checked in this experiment will include ±2.5,±5, and ±10. This leads to the experimental design described in Figure 2. Additionally, the standard takes on values of 30,40,50,60,70. This results in 320 unique triads for which to simulate responses.

The sample size was varied to determine a sufficient sample size for studies with a similar experimental design. In the simulations used for this study, number of responses per triad will be set to N=25,50,100,200, and 300.

#### Monte Carlo simulated responses

For this validation, it is assumed that the values of the characteristic used in the experimental value are understood to map onto the perception of the strength, $S_{i}=\psi_{i}$. For each triad, the response will be a Bernoulli distributed random variable where X=1 indicates that the participant selected test1 as the more different test. The probability of selecting test1, $p_{test1}$, is defined as

(13) ∑ptest1=ΦσDD(f(ψstand-ψtest1)-f(ψtest2-ψstand)).

The probability of selecting test1 relies on the specification of the discriminal dispersion, $\sigma_{DD}$, and the scaling function f. Both of these are varied in the simulations. It should be noted that here, the discriminal dispersion is the standard deviation of the Gaussian describing the perception of the difference of differences, whereas the discriminal dispersion that Thurstone defines is the standard deviation of the Gaussian describing the perception of the stimuli. However, the discriminal dispersion specified here is simply twice the discriminal dispersion defined by Thurstone. For the remainder of this article, the discriminal dispersion will refer to the standard deviation of the Gaussian in Equation (13).

#### Variables of the perception

The discriminal dispersion is a direct result of the JND. The JND is defined as the minimum difference needed to identify a difference 75% of the time. For these simulations, four differences in *S*, $d_{JND}=1.25,2.75,4.25,5.75$, will be considered a JND. Translating these values to the discriminal dispersion using the z-score (i.e., the inverse of the cumulative Gaussian (4)), for the 75% percentile,

(14) $$\frac{d_{JND}}{2\sigma_{DD}}=z_{0.75}.$$

$\sigma_{DD}$ takes on values of 3.65,8.03,12.41, and 16.79, respectively. The JND value is not likely to be known before an experiment takes place, so this value is varied to show how the model responds to data coming from a harder or easier task.

The underlying scaling function, f, indicates the existence of diminishing or increasing returns. This function is to be approximated by the MLE directly and should correspond to the significance and sign of the coefficient evaluated in the hypothesis test. Five functions were used to simulate the responses, three concave, one convex, and the linear relationship. The three concave relationships were selected to show the effect of curvature on the MLE's ability to approximate the correct function. Specifically, the following functions are used:

• Concave (circle): $f\left(x\right)=\sqrt{1-{\left(x-1\right)}^{2}}$,
• Concave (root): $f\left(x\right)=\sqrt{x}$,
• Concave (sine): f(x)=sin(x),
• Convex: $f\left(x\right)=x^{2}$,
• Linear: f(x)=x.

The functions are arbitrarily scaled such that they all intersect at (0,0) and (30,12) to suggest that a difference of 30 should be perceived as 12 units in the perceptual scale. The maximum of the perceptual scale is linearly related to the discriminal dispersion, so the scaling of the underlying functions is already varied.

#### Model selection

Since the size of the perceptual scale is linearly dependent on the standard deviation of the Gaussian used to compute the likelihood, only the perceived difference of the largest difference or the standard deviation needs to be estimated. It has been stated that, due to the linear relationship, the choice to model one or the other is arbitrary (Knoblauch et al., 2008). But there is actually a nuanced mathematical difference between fixing the maximum versus standard deviation. The former is a hard boundary (all of the learned values *must* fall under the maximum) while the latter is a soft boundary (all values that are too large become so improbable, the likelihood tends to zero for individual triads). Therefore, it is possible that setting one parameter over the other may produce an estimation closer to the ground truth. For that reason, MLE models that set the maximum, as is the case in the MLDS package (Knoblauch et al., 2008), as well as models that set the standard deviation were evaluated. If there was an analytic solution to the MLE, this would be irrelevant. However, due to the reliance on numerical optimization, particularly gradient descent, there is a chance that the gradient would be altered based on which parameter is set. As a result, there may be more local minima in which the optimizer would get “trapped,” leading to suboptimal and less stable fits.

To evaluate how the number of parameters the MLE estimates affects performance, three structures of the approximated function, $\hat{f}$, were used: two continuous cases, using the spline function, and the discrete case. In the continuous cases, the MLE will approximate 4 and 8 parameters. In the discrete case, 12 parameters will be estimated. In all cases, the perceived difference of the same stimuli was assigned to be 0.

Lastly, the inherent size of the perceptual scale, as determined by either the maximum or the standard deviation in the $\hat{f}$, was varied. The maximum was set at 1.2,12, and 120. These values correspond to standard deviation values of 0.146,1.46, and 14.6.

#### Performance metrics

For each combination of the factors above, a data set was generated. The hypothesis test was applied to the data sets and the significance and value of the $\beta_{2}$ coefficient were recorded. The MLE analysis was repeated 10 times with different, random initial conditions where all random values were uniformly distributed between (0,1). The average output was analyzed further.

The performance of the MLE was evaluated by two goodness-of-fit measures. The first is the negative log-likelihood used in optimization, defined in Equation (9). The second performance metric considered is how closely the approximated function, $\hat{f}$, matches the true function, f. This is quantified by the RMSE between the two,

(15) $$RMSE=\sqrt{\frac{1}{1000}\sum {\left(f\left(x\right)-\hat{f}\left(x\right)\right)}^{2}},$$

calculated by taking 1000 equidistant values in [0,1] and applying them to the scaled f and $\hat{f}$ such that the domain is [0,1].

### Predictions

The goal of this work is to evaluate the efficacy of significance testing and MLE approximation to understand the perception of varying sizes of differences. The following predictions were evaluated using the significance testing and MLE output for the simulated data:

P1: The significance testing can correctly identify if the underlying function is concave, convex, or linear.
P2: The MLE can model underlying functions.
P3: NLL is related to the RMSE of the underlying function and approximated function.

The relationship between the standard deviation and the maximum of the perceptual scale was evaluated, as well as if the choice of setting either results in lower negative log-likelihood or error. The effect of the size of the estimated perceptual scale and the effect of sample size were also investigated.

### Results

Simulated responses were analyzed using the proposed method. Both the output from significance testing and the MLE approximation of the underlying scaling function are analyzed.

#### Significance testing results

**Figure 3.**
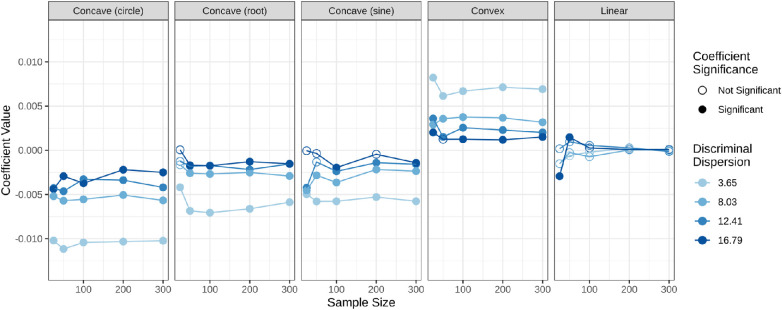
Value and significance of the $\beta_{2}$ coefficient in Equation (11). Significance is determined at the α=0.05 level.

To evaluate whether the value of coefficient $\beta_{2}$ in Equation (11) correctly identifies the concavity of the true underlying function f, the value and significance of the coefficient were analyzed. The values of the coefficients for each underlying scaling function, plotted by the sample size and underlying discriminal dispersion, can be seen in Figure 3. The concave functions correctly produced statistically significantly negative coefficients at the α level of the 0.05 value, although most coefficients would remain significant at lower alpha levels (e.g., α=0.01,0.001). Similarly, the convex function produced significantly positive coefficients and the linear function produced insignificant results with sufficient sample size.

The smaller the discriminal dispersion, the more extreme the coefficient in the nonadditive conditions. The largest discriminal dispersion required the largest sample size to produce nonsignificant responses in the linear condition and significant results in the nonlinear conditions, to some extent. This is consistent with the interpretation that a higher discriminal dispersion indicates more similar stimuli and thus a harder task.

#### MLE results

**Figure 4.**
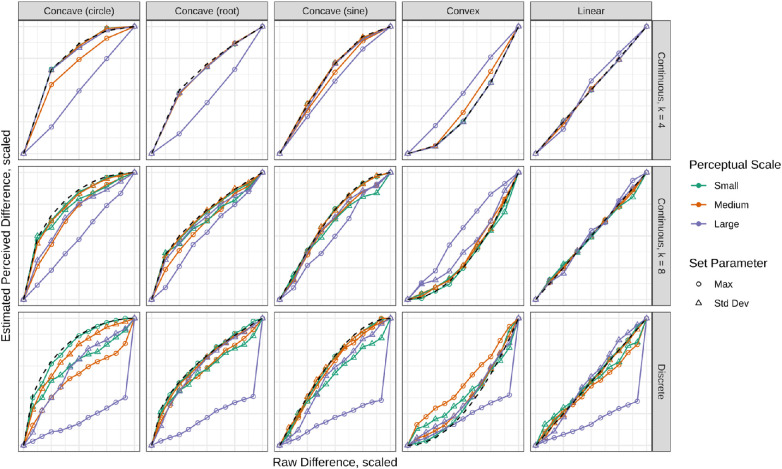
Approximated functions by the MLE. Results are summarized across sample sizes for a discriminal dispersion of 12.41 and sample size of 300. The true underlying functions are shown in a black dashed line. The three MLE models correspond to the rows of plots where k is the number of learned parameters. The *x*-axis is arbitrarily scaled and the *y*-axis is scaled such that all functions end at (0,0).

To evaluate how well the MLE produced the underlying scaling function, the output was plotted with the true function for comparison. These results can be seen in Figure 4. This plot only shows the results from sample size, N=300, but the results were consistent across other sample sizes. In general, the MLE can approximate the underlying function under certain choices for model selection. In particular, setting the maximum produces poorer fits, as seen by comparing the lines connecting circular markers to the true function. The larger perceptual scale ($max_{f}=120$ or $\sigma_{f}=14.6$), represented in purple, also performs worse. Overall, the MLE with fewer learned parameters produced a function that was closer to the ground truth within the combinations of set parameter and size of perceptual scale.

**Figure 5.**
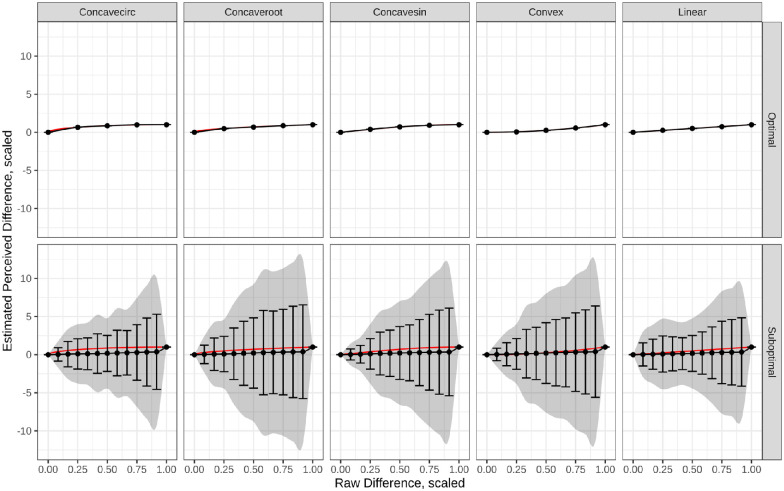
The 95% confidence intervals around the learned models are shown for the most and least optimal models. The optimal model (top row) corresponds with estimating only four parameters, setting the standard deviation, and a small perceptual scale. The suboptimal model corresponds to the discrete case where the maximum is set, and the perceptual scale is large.

Confidence intervals were omitted in Figure 4 to decrease visual clutter. However, the confidence intervals of the best- and worst-performing models are compared in Figure 5. Here, the shaded region shows the 95% confidence interval around the average learned model. Additionally, error bars indicate the confidence interval around the learned parameters. The models share a common scale to compare the variability in the learned models. This plot suggests that the model selection choices that result in a better-fitting model also produce more stable solutions.

**Figure 6.**
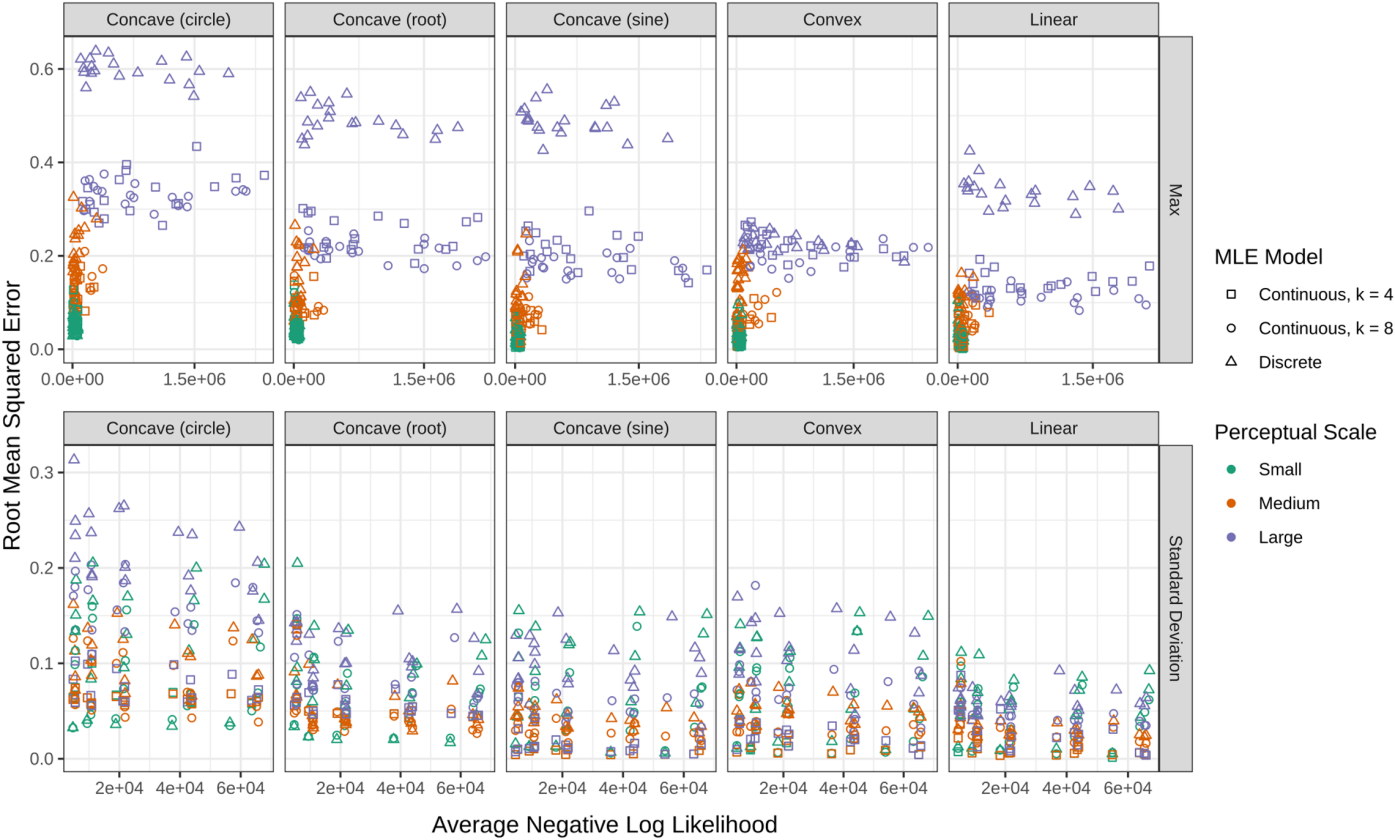
The relationship between RMSE and NLL based on the true underlying function (columns) by which parameter is set in the MLE (rows). The *x*- and *y*-axis ranges are both larger in the plots from the MLE with a set value for the maximum.

The NLL and RMSE for each model are compared in Figure 6. The average NLL was used to compare the NLL from different sample sizes. Setting the standard deviation versus the maximum produced lower RMSE and NLL values. Neither row shows a strong relationship between the NLL and the RMSE. If setting the maximum, a large perceptual scale produces worse RMSE and NLL. The hardest underlying function for the MLE to approximate appears to be the circular function regardless of setting the standard deviation or maximum.

**Figure 7.**
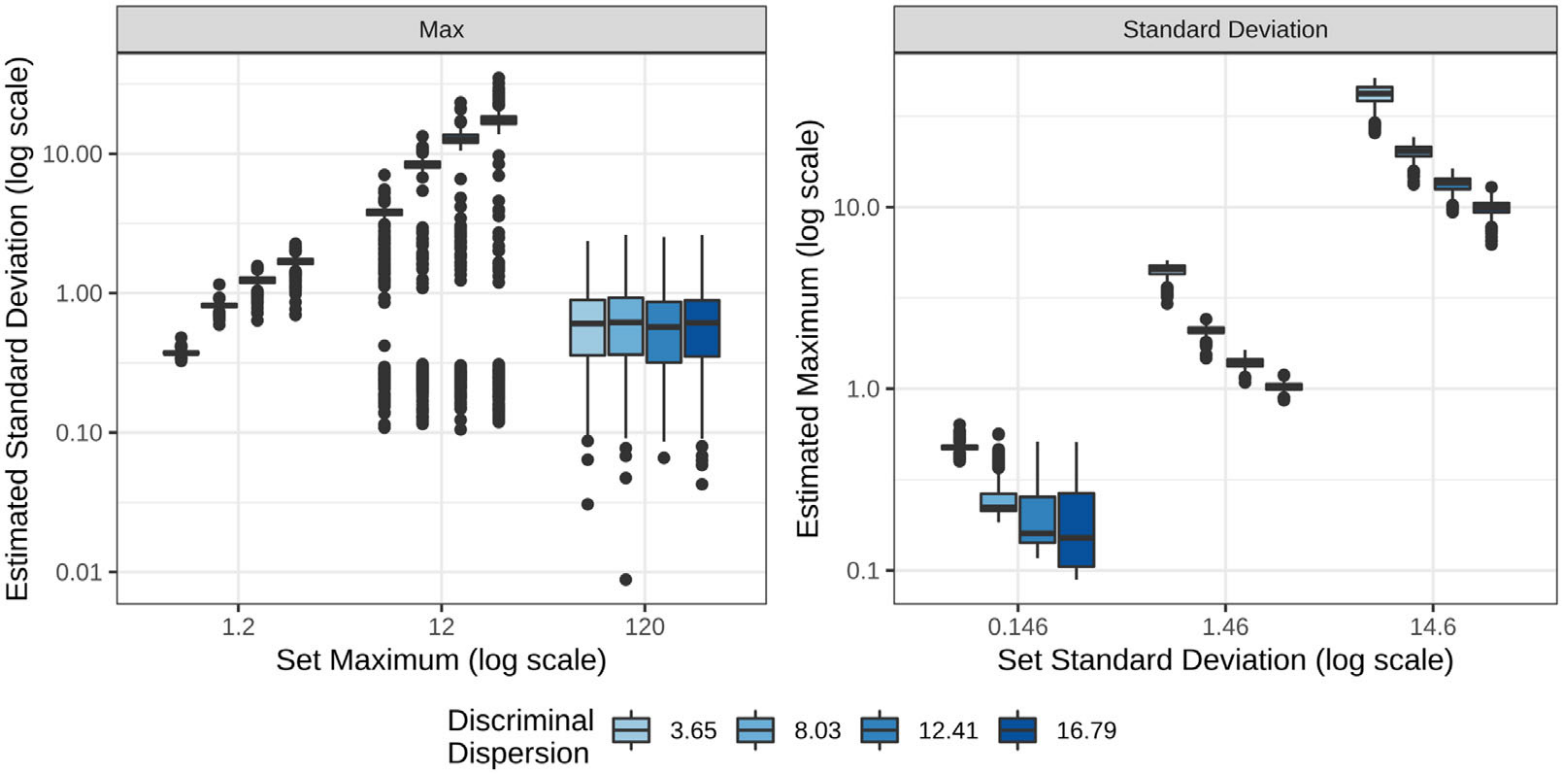
The distribution of the learned parameter when setting the other in the MLE approximation. Results are aggregated across all variables except the size of the perceptual scale, shown on the *x*-axis, and the discriminal dispersions, shown by the color of the boxplots.

The relationship between the maximum value of the perceptual scale and the standard deviation used in the MLE should be linear, which is why only one needs to be approximated. This relationship is summarized in Figure 7, where the left plot shows the standard deviations learned when setting the maximum, and the right plot shows the learned maximums by set standard deviations. In both plots, results are aggregated across sample sizes, model types, and underlying functions. There is a linear relationship between the estimated maximum by the set standard deviation within levels of the discriminal dispersions. This relationship is seen in the first two levels of the set maximum, but the estimated standard deviation drops when the perceptual scale is too large.

**Figure 8.**
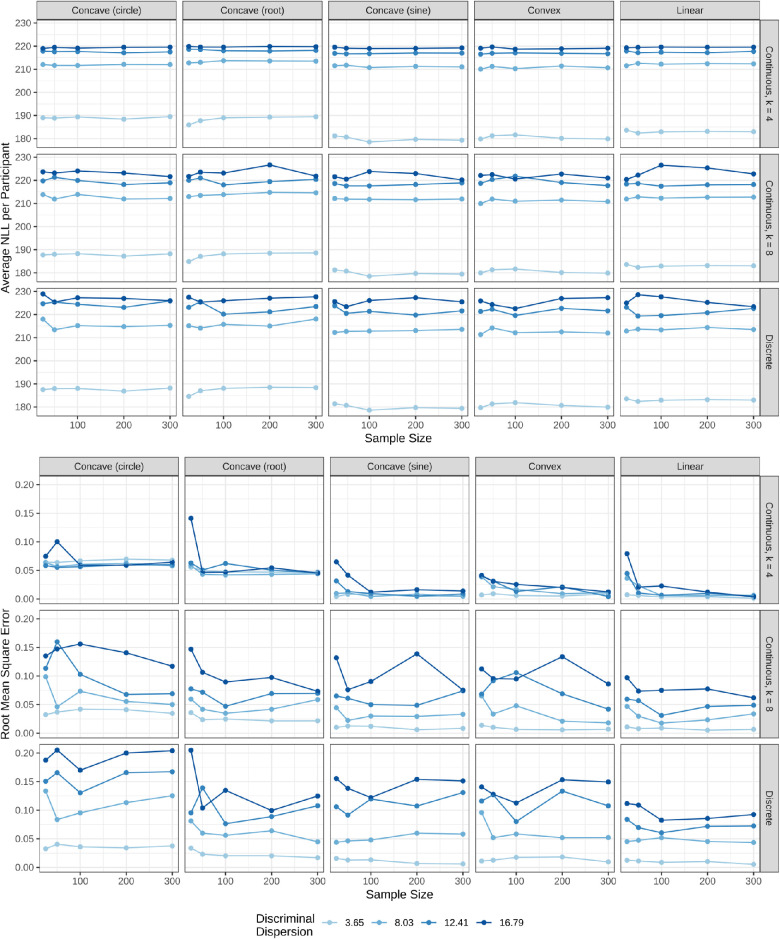
The average NLL per participant and RMSE of the MLE that sets the standard deviation to a medium-sized perceptual scale.

Lastly, the effect of sample size on the MLE approximation was evaluated. Both the NLL and RMSE are plotted based on the model selection, underlying scaling function, and discriminal dispersion. This plot includes only the results from MLE output when setting the standard deviation to 1.46, or a medium-sized perceptual scale. The results are shown in Figure 8. The average NLL is fairly robust to sample size, but the RMSE tends to decrease and level off with sufficient sample size (N>50), especially with fewer estimated parameters. This plot also confirms that, in general, the circular underlying scale function was harder for the MLE to approximate with more estimated parameters.

### Discussion

The proposed analysis to evaluate the additivity assumption in traditional MDS techniques was validated using simulated responses with and without additivity. Three predictions were made regarding the ability of significance testing and MLE modeling to return results consistent with the ground-truth conditions under which the data were simulated. Additionally, claims about the relationships of the parameters in the MLE and the effect of sample size were evaluated.

Prediction P1, that the significance testing can correctly identify the concavity of the underlying function, is supported. Figure 3 shows that the coefficient for the average difference in the triad, $\beta_{2}$ in Equation (11), was significantly negative for data simulated with a concave scaling function. This indicates that in triads with a larger average difference, it is more difficult for participants to correctly identify the more different test stimulus. This result is consistent with the ground truth, where diminishing returns was represented by a concave scaling function. Similarly, the $\beta_{2}$ coefficients are significantly positive for data generated with a convex scaling function. This supports that as the average difference increases, the differences are easier to distinguish, which is consistent with the underlying ground truth of increasing returns. Lastly, the $\beta_{2}$ coefficients are not significant when data are generated with additivity of differences, except in the case of higher discriminal dispersion and low sample sizes.

Prediction P2, that the MLE can model the true underlying scaling functions, is generally supported. The ground truth and approximated functions are seen in Figure 4. In general, the MLE performed best with fewer parameters, small or medium perceptual scales, and when setting the standard deviation, shown as orange and green lines denoted by triangular markers. This supports that the true underlying functions can be closely approximated with this model selection.

The third major prediction, P3, that the NLL is related to the RMSE of the approximated ground-truth function, was not supported. Figure 6 shows the lack of a positive correlation between the two performance metrics. Models where the standard deviation is set, meaning the maximum value is approximated, resulted in lower NLL and RMSE, indicating this is the superior choice for which parameter to set. However, even in this case, represented by the bottom row of plots, there is not a relationship between NLL and RMSE. This suggests that a sufficiently low NLL does not guarantee the approximated function will match the true function. However, these results, combined with the success of the optimal MLE approximation regime discussed above, do support that the function can be correctly approximated.

As consequence of the lack of relationship between RMSE and NLL, the applicability of likelihood-based performance metrics is called into question. Consider the Akaike information criterion (AIC), which is defined as

(16) $$\begin{array}{c} AIC=2k-2\ln(\hat{L})\; \end{array}$$

where k is the number of parameters in the model and $\ln(\hat{L})$ is the log-likelihood. This is a linear transformation of the NLL and, therefore, also would not be correlated with how well the model predicted the ground truth. Another common metric used is deviance, which is twice the difference of the log-likelihood of a saturated model and the log-likelihood of the model being evaluated. Again, this is a linear transformation of the NLL and therefore not related to how closely the model being evaluated lies to the ground truth. In practice, the ground truth is unknown, so reliance on only log-likelihood-based performance metrics may be misleading if the performance metric is intended to show how well the learned model approximates ground truth.

Previous analysis using MLE for difference scaling to produce a scale of stimulus strengths opts to set the maximum value and approximate the standard deviation (Knoblauch et al., 2008). Although unlikely, this claim may be true for perceptual scales of stimuli; however, when modeling the underlying scaling function, setting the standard deviation produces lower NLL and RMSE compared to the true function. Additionally, when setting the standard deviation, the estimated maximum does increase linearly with the set value while this relationship is not maintained for large perceptual scales when setting the maximum and learning the standard deviation. As seen in Figure 7, the estimated value of the standard deviation drops with the largest value of the fixed maximum. This likely explains the higher RMSE and NLL seen in Figure 6 for the large perceptual scale when fixing the max.

Lastly, the effect of sample size on NLL and RMSE is evaluated. Figure 8 shows that the NLL is robust to the sample size, while the RMSE is affected. The NLL is more driven by the underlying discriminal dispersion than the sample size. In general, the RMSE decreases with the sample size, which is to be expected. However, as the number of parameters increases in the MLE, the RMSE also tends to increase. This is consistent with the trend in Figure 4 that the MLE model with fewer parameters approximated the underlying function more closely. From the top row in Figure 8, which is the optimal model for MLE, the RMSE starts to level off with a sample size greater than 100, but this is moderated by the underlying discriminal dispersion.

## Validation study without assuming a known perceptual scale

The previous study assumed that a perceptual scale is known so stimulus strengths can be described by their perceived strengths, $S_{i}=\psi_{i}$. This scale often is not known at the time of the experiment, and even a known scale likely depends on assumptions about the additivity of difference perception. For that reason, the MLE modeling of $\hat{f}$ was further validated on data simulated where the relationship between absolute strengths, $S_{i}$ and perceived strengths, $\psi_{i}$, is nonlinear.

### Methods

The same experimental triads were used as in the previous study. The main difference is in the Monte Carlo simulation of the data. The overall framework remains the same, with the addition of a perceptual scale, g, which maps from stimulus strength to perceived strength, $g\left(S_{i}\right)=\psi_{i}$. Thus, Equation (13) is updated to be the following, similarly described by Krantz (1967),

(17) ptest1=ΦσDD(f(|g(Sstand)-g(Stest1)|)ptest1=-f(|g(Stest2)-g(Sstand)|)).

The perceptual scale and true scaling function are manipulated here.

#### Perceptual scale

Three perceptual scales are used in this study: one concave, one convex, and one linear. The linear perceptual scale essentially replicates the first study for comparison. Specifically,

• Concave: $g\left(x\right)=\sqrt{x}$,
• Convex: $g\left(x\right)=x^{2}$,
• Linear: g(x)=x.

These functions are selected to determine if the concavity of the perceptual scale impacts the MLE's performance in a systematic way. The output of the g functions is used to calculate the differences, $|g\left(S_{i}\right)-g\left(S_{j}\right)|$, which are then scaled to be between 0 and 1.

#### True scaling function

Only three of the five previously tested functions were used in this study. They take the same form as the g functions. The inputs to the f functions were previously scaled, so that both the domain and range are [0,1].

#### Fixed factors

The underlying discriminal dispersion, $\sigma_{DD}$, is calculated for each level of the perceptual scale, such that on average, a difference of differences of 5 is considered a JND. Only the continuous MLE with 4 estimated parameters is used and the standard deviation is fixed to be 0.25, leaving the maximum value to be estimated. These settings were chosen because of their superior performance in the previous study.

#### Performance metrics

For each combination of g and f, 10 data sets are generated and analyzed by the MLE once. From these fits, the RMSE is calculated. Additionally, a new performance metric is considered. The MLE's accuracy in predicting participants’ responses is considered here. The NLL is not used in this study, as it was not seen to correlate with RMSE, which motivates the investigation of accuracy as a goodness-of-fit measurement.

#### Predictions

Two predictions are made here:

P4: The ability of the MLE to approximate $\hat{f}\left(x\right)$ will decrease with a nonlinear g(x).
P5: The RMSE will be related to the accuracy across all levels of g(x).

### Results

**Figure 9.**
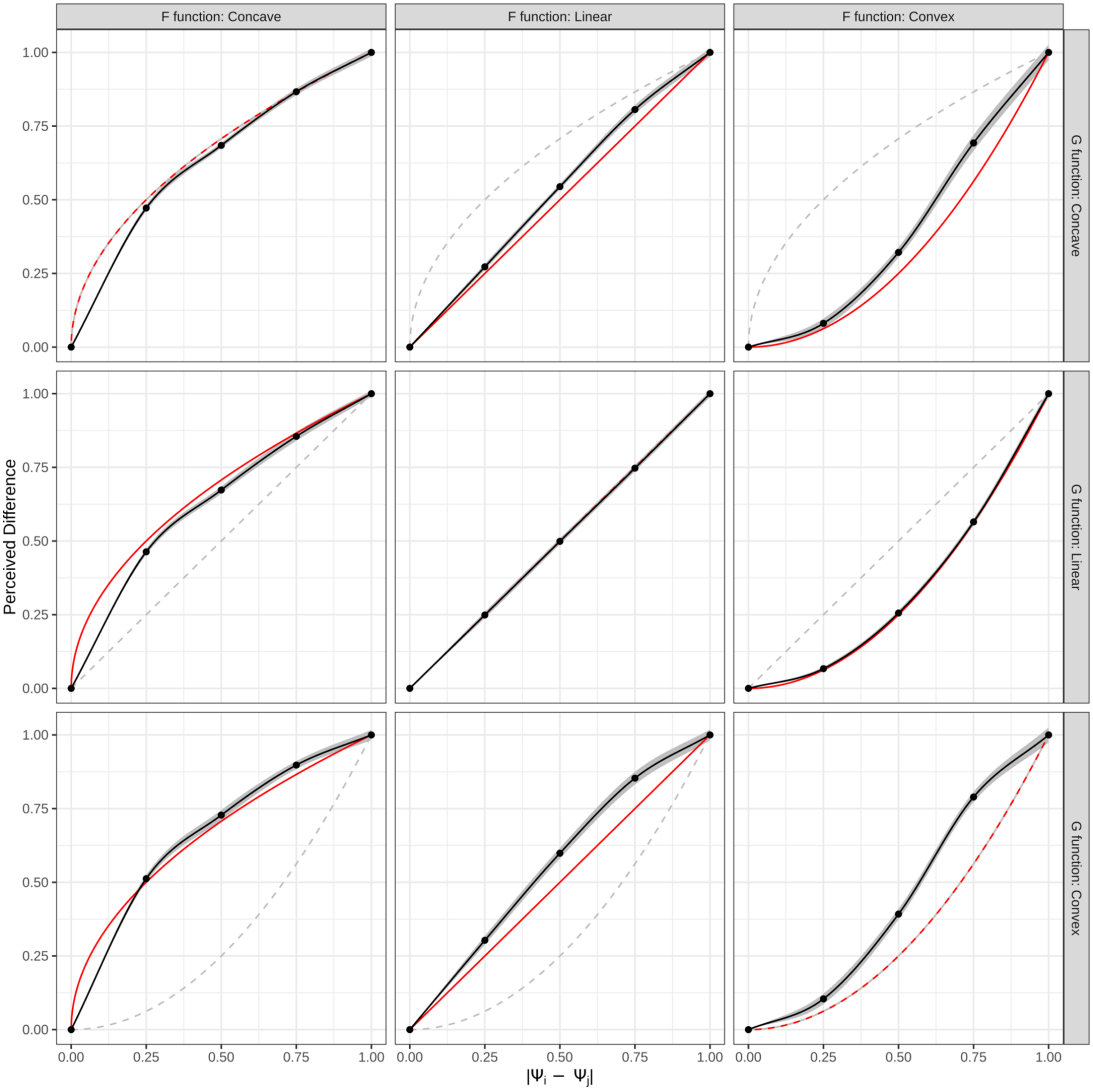
Approximated $\hat{f}\left(x\right)$ functions are shown in black lines with the MLE-estimated parameters shown as black points; these are averaged across the 10 repetitions. The 95% confidence intervals are shown around the models as a shaded gray region. The red solid line is the true f(x) function and the dashed gray line is the perceptual scale, g(x), which does not share the same *x*-axis as f(x) and $\hat{f}\left(x\right)$.

The fits of the MLE are seen in Figure 9. The MLE was able to produce a reasonably close fit to f even in the presence of a nonlinear g. The MLE is able to match the global concavity in most cases, with some exceptions on the larger side of $\hat{f}$. The regions where the concavity of $\hat{f}$ does not match f are relatively small and appear only on the larger side of the domain, meaning that the two values, from $\psi_{i}=g\left(S_{i}\right),\psi_{j}=g\left(S_{j}\right)$, were far apart. This is where the nonlinear perceptual scale is expected to have the biggest effect, as a linear approximation of g across a wide range will produce a worse fit than a linear approximation across a more narrow range.

**Figure 10.**
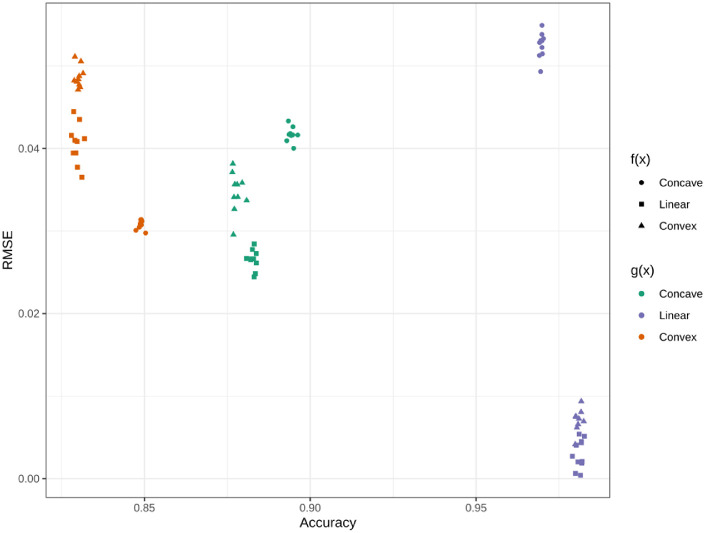
RMSE and accuracy of the MLE fits to predict responses for the 10 data sets generated for every combination of the perceptual scale, g(x), and the true scaling function, f(x).

The performance metrics and their relationship are seen in Figure 10. Interestingly, the RMSE is high for the MLE fit to data generated with a linear perceptual scale but concave scaling function. This is despite a high accuracy for predicting participant responses. The RMSE is high, despite the $\hat{f}$ mapping fairly close to the f but consistently being a slight underapproximation. Overall, there was a significant negative relationship between RMSE and accuracy, R=-0.541,t(88)=-6.037,p<0.001, supporting the use of accuracy as a goodness-of-fit measurement for MLE models.

### Discussion

Other than the linear perceptual scale and concave scaling function, *P4: the ability of the MLE to approximate $\hat{f}\left(x\right)$ will decrease with a nonlinear g(x)* holds. This is demonstrated by inspection of Figure 9 and the lower RMSE of the purple points in Figure 10. The final prediction, *P5: The fit between f(x) and $\hat{f}\left(x\right)$ will be related to the accuracy across all levels of g(x)*, is also supported.

Ultimately, the use of a nonlinear perceptual scale, simulating when the true perceived strengths of stimuli are unknown or accounted for, showed support for the use of MLE to model f. Despite the lack of consideration for g in the approximation of f, the MLE produced $\hat{f}$s that fell close to the ground truth. The global concavity was maintained, with any errors happening in the last segment of the spline. Therefore, Prediction P4 is supported by this study.

Accuracy of the MLE was found to be an appropriate goodness-of-fit measurement, as NLL was not found to correlate with RMSE. Thus, Prediction P5 was correct. It has been suggested that the use of a simulated distribution of deviance may be used to evaluate the goodness of fit of an MLE model (Protonotarios et al., 2016); however, this claim is only appropriate if the MLE aims to model the entire perceptual process. This method was applied to these simulations but ultimately was too conservative, rejecting MLE approximations of f that had high accuracy and low RMSE. For future use of MLE to approximate the underlying scaling function, accuracy is recommended to evaluate the goodness of fit as it is correlated with RMSE.

## Application to empirical data sets

This analytical approach has recently been applied to color perception, providing strong evidence for the existence of diminishing returns in color perception (Bujack et al., 2022). This work used MOT, where triads were constructed at five discrete centers and the tests varied based on their differences. This approach is distinct from many MOT studies as it did not include all possible triads from the stimulus set, but rather a subset of 320. Designing the experiment in such a way allowed for the balancing of differences in the data set so the difference scaling method would not be biased toward more frequent differences. Between 250 and 300 responses per triad were recorded using a crowdsourced platform, and from these data, the difference scaling method was compared to estimating the perceptual scale alone. The results indicate that the scaling of differences is essential to accurately model human color perception.

Here, we demonstrate the applicability of our method to previously collected data for comparison to estimating the perceptual scale alone. In all cases, we first estimate the perceived strengths using MLDS and then the difference scaling function on top of the perceptual scale. For both estimations, we compare the predictive power of the model.

### Potential nonadditivity in the watercolor effect

Devinck and Knoblauch (2012) used MOT to investigate the watercolor effect (WCE), an optical illusion where whitespace surrounded by a colored polygon can appear to have a light chroma. The original experimental design included 10 levels of the luminance of the interior color of the polygon, which created varying intensities of the WCE. Four participants were shown all possible combinations of the 10 stimuli in triads and were asked to indicate which of the tests were more similar to the standard.

A consequence of using all possible combinations of three stimuli selected from 10 levels is the unbalanced nature of the differences compared. For instance, if the standard is the second level of the independent variable, there is only one option for one test while there are eight for the other. As a result, there are many more small differences than large differences (e.g., there were 72 triads that contained a difference of 1, while only 2 contained a difference of 8). In order to use a more balanced data set with respect to the differences, we only used triads with the middle standards (Levels 4, 5, 6, 7) and no test was more than three levels beyond the standard. This resulted in 36 unique triads, each of which had 40 responses. Balancing the data with respect to differences is important as the log-likelihood weighs each data point equally. In the case of an unbalanced design, if the model does a poor job at approximating large differences, this will not have as large an impact on the NLL as if the model does a poor job approximating small differences.

**Figure 11.**
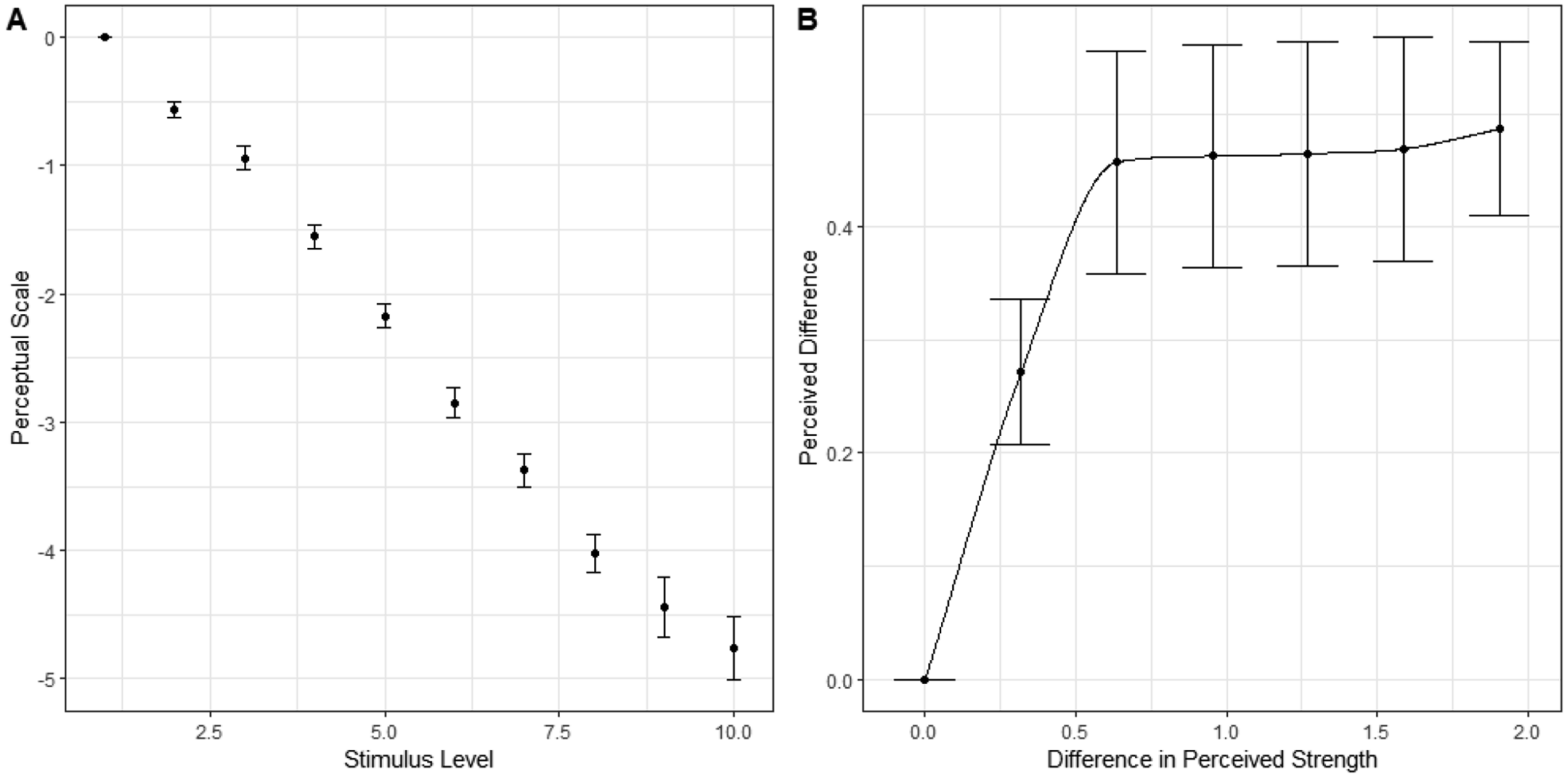
Average learned models of the perception of the WCE (Devinck & Knoblauch, 2012). (A) Shows the learned perceptual scale using MLDS while (B) shows the learned difference scaling function applied on top of the perceptual scale.

We used fourfold cross-validation to evaluate the generalizability of modeling the perceptual scale using MLDS and difference scaling using the estimated perceptual scale. The average perceptual scale and difference scaling function are seen in Figure 11. Modeling the perceptual scale achieved only a higher validation accuracy (86.14%±1.68% at the 95% confidence level) than the difference scaling model (76.11%±1.18%).

**Figure 12.**
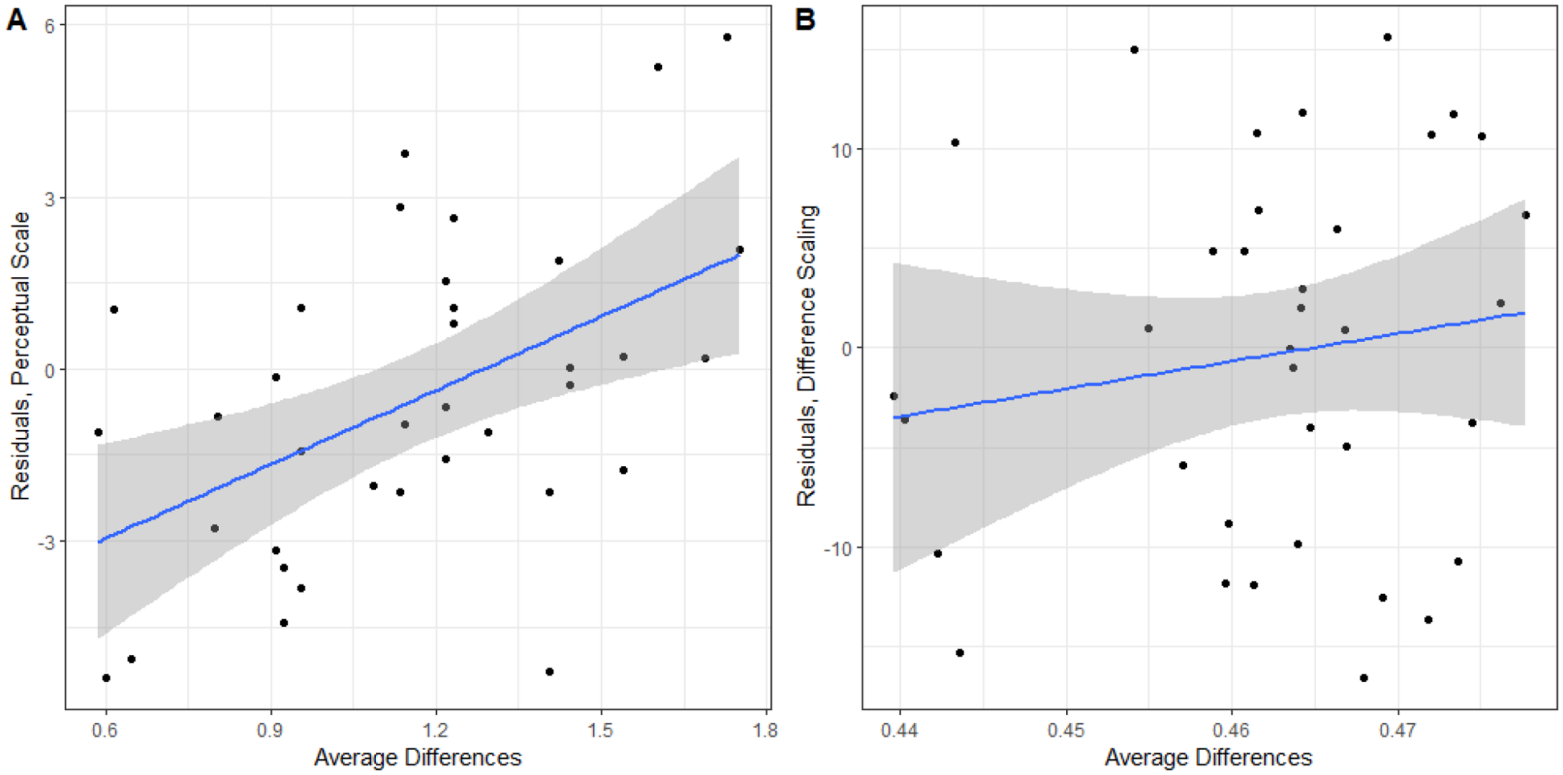
Residuals when predicting responses to WCE triads based on the average difference in the triad. Separate plots for the perceptual scale or the difference scaling models.

We also analyzed how well the model predicted responses across the average difference in the triad. If difference scaling is not necessary, there should not be a relationship between the average difference in the triad and the residual. The residuals of the predicted responses compared to observed are plotted against the average difference in the triad for both models in Figure 12. There is a significant linear trend for the perceptual scale model, R=0.505,t(34)=3.4076,p<0.005; however, this relationship does not exist for the difference scaling model, R=0.153,t(34)=0.9013,p>0.1. This indicates that, while the difference scaling model predicts responses worse overall, it does so in the same manner across all triads. The perceptual scale does well for the average triad but does not perform consistently across all triads and could be improved by taking into account the size of the differences in the triads. It may be the case that there were not enough triads or levels of differences to adequately characterize the nonadditivity in comparisons of the WCE, and further work with a balanced design with respect to the differences in the triads might be considered.

### Additivity in slant-from-texture

Aguilar et al. (2017) investigated the judgment of slant from two-dimensional images using MOT. Images were generated at 8 degrees of slant, giving a total of 56 unique triads judged by eight individuals. In an effort to balance the differences, as described above, we only analyzed triads where the standard was the fourth or fifth stimulus level. This resulted in 48 triads that were viewed an average of 48.8 times each.

**Figure 13.**
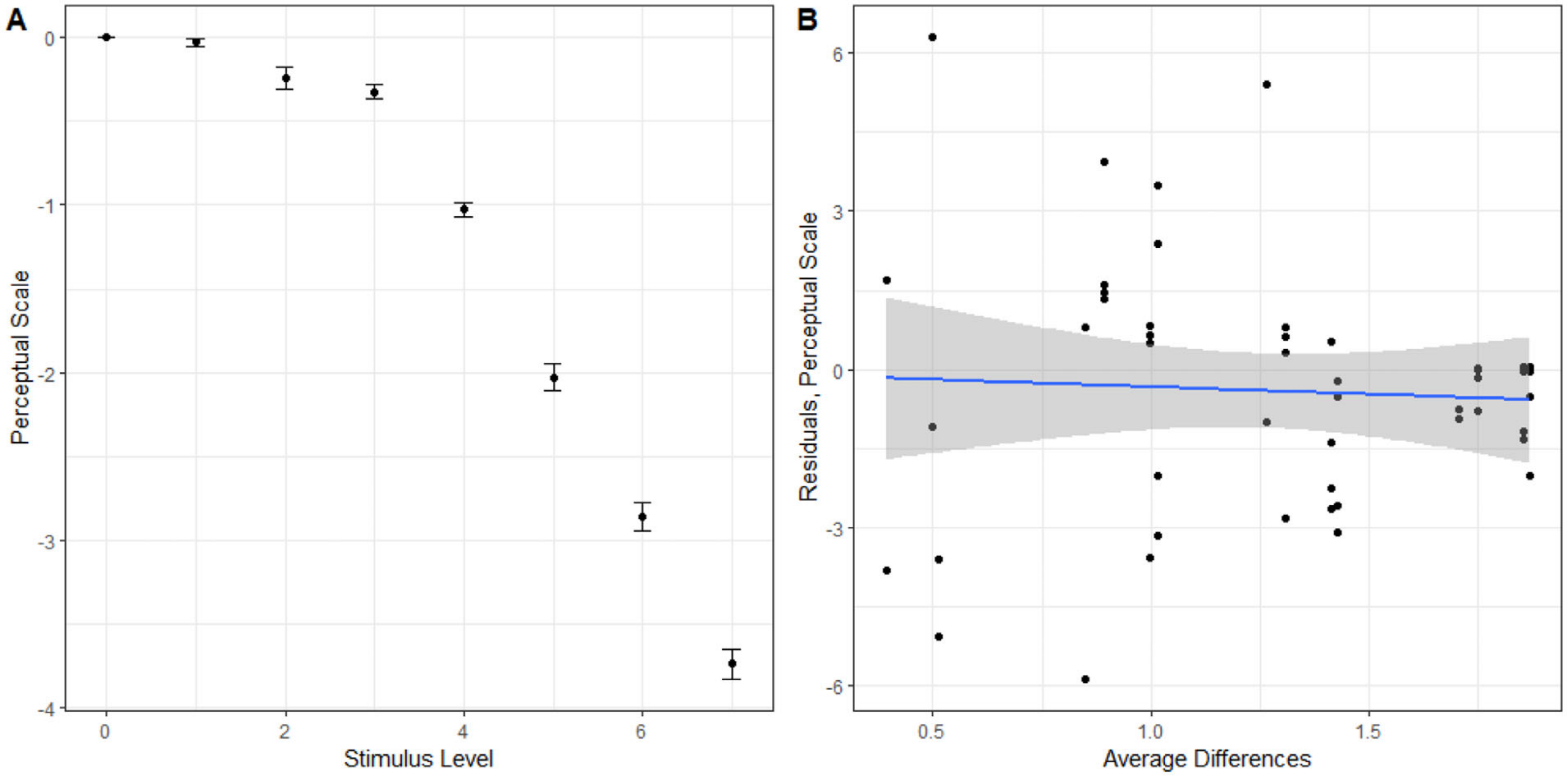
(A) Estimated perceptual scale based on slant-from-texture experiments (Aguilar et al., 2017). (B) Residuals of predicted responses based on average difference in the triad using the perceptual scale.

Performing the same cross-validation as described above, the average difference scaling model predicted responses much worse, 63.97%±0.62%, compared to only using the perceptual scale, 93.37%±0.46%. Additionally, there was no correlation between the average difference in the triad and the residual when predicting responses using only the perceptual scale, R=-0.052,t(46)=-0.35,p>0.1. The perceptual scale and residuals can be seen in Figure 13. Combined, these results suggest that adding the perception of small differences to estimate the perception of larger differences is appropriate for this perceptual task.

### Diminishing returns in color contrast judgments

Wiebel et al. (2017) estimated the perceived lightness of patches within a complex setting over several viewing conditions. Stimuli were varied by their lightness and embedded into an image. There were 10 levels of the stimuli; however, here we consider only the middle four and with differences limited to no more than three stimulus levels apart. This led to a total of 72 triads per viewing condition with approximately 50 responses per triad, or 3,600 trials per viewing condition.

For each viewing condition, we performed 10-fold cross-validation. In every viewing condition, the model without accounting for the size of the differences predicted participant responses approximately at chance level. The perceptual scale was most predictive in the low-transparency light viewing condition, 59.27%±1.61%. When differences were scaled, the predictive power increased to slightly above 70%. Overall, the increase when applying difference scaling was 18.95%±0.60%.

**Figure 14.**
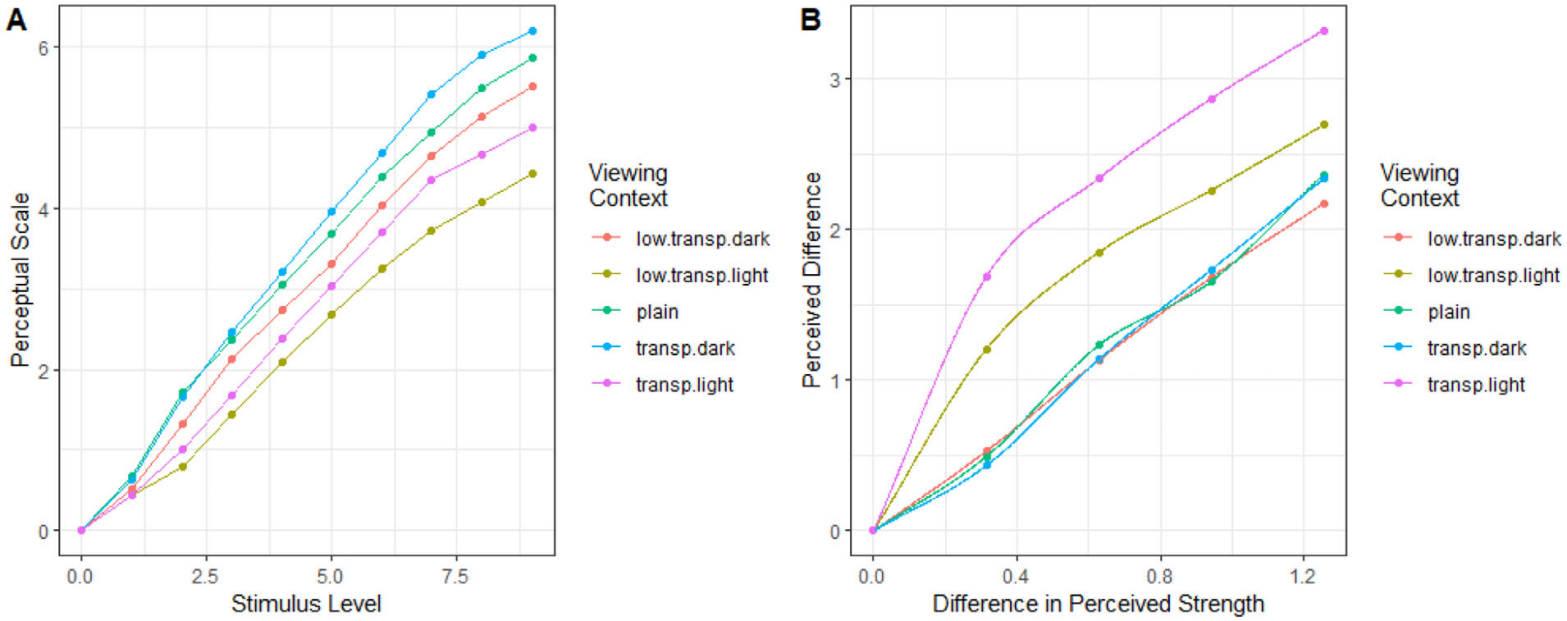
Learned perceptual scale and difference scaling functions for perceived lightness in a complex presentation (Wiebel et al., 2017). Stimuli were shown in five viewing conditions, indicated by color in each plot.

The learned models for all five viewing conditions are seen in Figure 14. On the left, we see the learned perceptual scale, while on the right, we see the learned difference scaling functions. There appears to be a slight curvature in the middle region for the plain viewing condition while, for the two light conditions, we see a dramatic effect of diminishing returns. This is perhaps unsurprising given the findings of Bujack et al. (2022). However, Wiebel et al. (2017) place the stimuli in a more complex presentation and thus the existence of diminishing returns was not guaranteed.

## Properties of psychophysical estimation procedures

When modeling psychophysical scales from sensory processes, certain conditions have been proposed (Krantz et al., 1971). Two of these conditions, or axioms, are testable: the ordering property and the six-point property. Maloney and Yang (2003) discuss both in the introduction to maximum likelihood for difference scaling. The ordering property requires that participants can reasonably order stimuli. This property is not inherent to the analysis method but rather the experimental design. Above, we have suggested using stimuli approximately 1 JND apart; this would result in a stimulus set with the most number of distinct stimuli along a restricted gamut of a quantifiable characteristic (e.g., lightness, slant). However, if the perceptual distance between stimuli is subthreshold, the ordering property does not hold, as $\psi_{i}\approx \psi_{j}$ might result in a participant considering $\psi_{i}>\psi_{j}$ even though $S_{i}<S_{j}$.

The second condition, the six-point property, is inherent to the analysis method. Maloney and Yang (2003) and Knoblauch et al. (2008) discuss this property and include a test for it using MOQ. We have not reformulated it for MOT. However, we can abstract it beyond MOT and MOQ to demonstrate that (1) the six-point property does not address the nonadditivity of small differences and (2) the theory underlying our method does not appear to violate this property.

For any three stimuli, $i<j<k,i^{'}<j^{'}<k^{'}$, suppose the observer perceives the difference between i and j to be less than the difference between $i^{'}$ and $j^{'}$. This can be denoted as

(18) $$\begin{array}{c} ij<i^{'}j^{'}.\; \end{array}$$

If the observer also judges

(19) $$\begin{array}{c} jk<j^{'}k^{'}\; \end{array}$$

it must follow that

(20) $$\begin{array}{c} ik<i^{'}k^{'}.\; \end{array}$$

If we assume additivity, we can see this simply by considering $\psi_{i},\psi_{j},\psi_{k},\psi_{i^{'}},\psi_{j^{'}},\psi_{k^{'}}$. From Equation (18), we know

(21) $$\psi_{j}-\psi_{i}>\psi_{j^{'}}-\psi_{i^{'}}$$

and from Equation (19), we know

(22) $$\psi_{k}-\psi_{j}>\psi_{k^{'}}-\psi_{j^{'}}.$$

If we can assume that small differences can be added to equal large differences, we can add Equations (21) and (22),

(23) $$\;\;\;\;\;\;\;\;\;\;\;\;\left(\psi_{k}-\psi_{j}\right)+\left(\psi_{j}-\psi_{i}\right)>\left(\psi_{k^{'}}-\psi_{j^{'}}\right)+\left(\psi_{j^{'}}-\psi_{i^{'}}\right),$$

which can be simplified to

(24) $$\psi_{k}-\psi_{i}>\psi_{k^{'}}-\psi_{i^{'}}.$$

This inequality is consistent with the judgment $ik>i^{'}k^{'}$.

Our method does not violate this property, and this property does not imply or check for nonadditivity of small differences. We can show that this property is preserved with a monotonic scaling function, f(x), that accounts for diminishing (or increasing) returns. From the monotonicity, it follows

(25) $$\;\;\;\;\;\;\;\;\;\;\;\;\;\psi_{j}-\psi_{i}>\psi_{j^{'}}-\psi_{i^{'}}\Rightarrow f\left(\psi_{j}-\psi_{i}\right)>f\left(\psi_{j^{'}}-\psi_{i^{'}}\right).\;\;\;\;$$

From the judgment of i,j and $i^{'},j^{'}$, we know

(26) $$f\left(\psi_{j}-\psi_{i}\right)>f\left(\psi_{j^{'}}-\psi_{i^{'}}\right)$$

and from the judgment of j,k and $j^{'},k^{'}$, we know

(27) $$f\left(\psi_{k}-\psi_{j}\right)>f\left(\psi_{k^{'}}-\psi_{j^{'}}\right).$$

Due to the relationship in (25), we know

(28) $$\psi_{j}-\psi_{i}>\psi_{j^{'}}-\psi_{i^{'}}$$

(29) $$\psi_{k}-\psi_{j}>\psi_{k^{'}}-\psi_{j^{'}}.$$

Therefore, if we add the smaller differences and then scale, we get

(30) $$\;\;\;\;\;\;\;\;\;\;\;\;\;\;\psi_{k}-\psi_{i}>\psi_{k^{'}}-\psi_{i^{'}}\Rightarrow f\left(\psi_{k}-\psi_{i}\right)>f\left(\psi_{k^{'}}-\psi_{i^{'}}\right),\;\;$$

which is consistent with the six-point property despite the presence of nonadditivity. As a result, observations that comply with the six-point property do not imply additivity of small differences.

It is possible that the likelihood of violating the six-point property, defined as $\Lambda_{6}$ in the previous work, is impacted by the existence of diminishing returns (Maloney & Yang, 2003; Knoblauch et al., 2008). The previous authors took great care to explain the effects of a stochastic observer, especially when the differences are very similar. With a monotonic scaling function, particularly one that is concave as in the case of diminishing returns, larger differences are perceived as smaller than the addition of smaller differences. Therefore, $\Lambda_{6}$ may be related to the size of $ij,jk,ik,i^{'}j^{'},j^{'}k^{'},i^{'}k^{'}$. However to test this relationship, quadruples with only (relatively) small differences and only (relatively) large differences would need to be compared. The number of quadruples that contains only large differences will be much smaller than the number of quadruples with only small differences, as there will almost certainly be a gamut restriction of the quality that is being manipulated (e.g., slant, image compression, or lightness). We agree that expanding this work to further investigate the relationship of $\Lambda_{6}$ and the nonadditivity of small differences may be promising, but it is not immediately clear how to test such a relationship. We leave this question open for future work.

## Conclusions

MLE can be used to approximate an underlying scaling function with the optimal combination of parameters, even when the perceived strengths of stimuli are unknown. Specifically, fewer parameters should be learned and the standard deviation of the underlying cumulative normal distribution should be fixed while the maximum value in the function should be estimated. The perceptual scale should not be too large, instead opting for an approximate JND to be 0.1 or 1 units in the perceptual space. The size of the perceptual scale will dictate the value selected for the standard deviation in the cumulative normal distribution. For instance, if estimating an underlying function that is scaled such that the input of 0.1 should lead to a 75% accuracy rate, it is trivial to solve for the corresponding standard deviation,

(31) $$\begin{array}{ccc} \Phi_{\sigma_{f}}\left(0.1\right) & \;= & 0.75\\ \frac{0.1}{\sigma_{f}} & \;= & \Phi^{-1}\left(0.75\right)\;\\ \sigma_{f} & \;= & \frac{0.1}{z_{0.75}}\\ \sigma_{f} & \;\approx & 0.146 \end{array}$$

where $\Phi^{-1}$ is the inverse cumulative normal distribution with μ=0,σ=1 and $z_{0.75}$ is the *z*-score of the 75th percentile. The value that a JND should correspond to in the perceptual scale can be selected, but the results suggest that too large of a value may result in suboptimal results.

This analysis can be used with a quantifiable characteristic of stimuli that has a way to draw samples such that the differences are perceived roughly the same regardless of the values of the characteristic. For example, this analysis will work if a $d_{1}$ is perceived the same regardless of whether the standard is 50 or 70. If such a scale is unknown, the MLE still produces functions with overall concavities matching the ground-truth functions. However, the accuracy of the model to predict participant responses is suggested to determine whether the model achieved sufficient goodness of fit.

The first results presented here are based on Monte Carlo simulated responses to triads. Additionally, we have applied our method to a study of diminishing returns in the perception of grays. Bujack et al. (2022) found that even after using a similar MLE approach to model the transformation from absolute strength to perceived strength, modeling a difference scaling function increased accuracy in predicting responses to triads. This significant increase in accuracy suggests that this method is successful in an empirical setting, and the work included in the present study lends confidence to the method's ability to capture the correct shape of the scaling function.

We also applied our method to data from other studies using MOT. We saw that in the case of the WCE, there is weak evidence for nonadditivity based on the study by Devinck and Knoblauch (2012). When modeling the perception of slant-from-texture, we saw strong evidence for additivity in difference perception based on work by Aguilar et al. (2017). Lastly, our method found strong evidence for diminishing returns in lightness comparisons using data collected by Wiebel et al. (2017). These results highlight that not every perceptual task is likely to have additivity, which supports the need to check this assumption.

Analytical methods to construct perceptual scales based on Thurstone's Case V, either using Torgeron's method or MLE, for judgments of large differences have failed to produce meaningful insights (Protonotarios et al., 2016) and have been identified as controversial (Maloney & Yang, 2003). This work directly addresses how to compare small and large differences in a quantitative characteristic without assuming large differences can be understood as the sum of small differences. The ability to understand whether the sum of small differences over- or underestimates large differences allows for a rigorous investigation of Thurstone's theory of comparative judgments.
